# Effects of bolus injection duration on perfusion estimates in dynamic CT and dynamic susceptibility contrast MRI

**DOI:** 10.1007/s10334-022-01038-y

**Published:** 2022-09-17

**Authors:** Jonathan Arvidsson, Göran Starck, Kerstin Lagerstrand, Doerthe Ziegelitz, Oscar Jalnefjord

**Affiliations:** 1 Department of Medical Radiation Sciences, Institute of Clinical Sciences, Sahlgrenska Academy, University of Gothenburg, Gothenburg, Sweden; 2 Department of Medical Physics and Biomedical Engineering, Sahlgrenska University Hospital, Blå stråket 7, vån 2, 413 45 Gothenburg, Sweden; 3 Department of Radiology, Institute of Clinical Sciences, Sahlgrenska Academy, University of Gothenburg, Gothenburg, Sweden; 4 Department of Neuroradiology, Sahlgrenska University Hospital, Gothenburg, Sweden

**Keywords:** Perfusion imaging, Cerebral blood flow, Cerebral blood volume

## Abstract

Estimates of cerebral blood flow (CBF) and tissue mean transit time (MTT) have been shown to differ between dynamic CT perfusion (CTP) and dynamic susceptibility contrast MRI (DSC-MRI). This study investigates whether these discrepancies regarding CBF and MTT between CTP and DSC-MRI can be attributed to the different injection durations of these techniques. Five subjects were scanned using CTP and DSC-MRI. Region-wise estimates of CBF, MTT, and cerebral blood volume (CBV) were derived based on oscillatory index regularized singular value decomposition. A parametric model that reproduced the shape of measured time curves and characteristics of resulting perfusion parameter estimates was developed and used to simulate data with injection durations typical for CTP and DSC-MRI for a clinically relevant set of perfusion scenarios and noise levels. In simulations, estimates of CBF/MTT showed larger negative/positive bias and increasing variability for CTP when compared to DSC-MRI, especially for high CBF levels. While noise also affected estimates, at clinically relevant levels, the injection duration effect was larger. There are several methodological differences between CTP and DSC-MRI. The results of this study suggest that the injection duration is among those that can explain differences in estimates of CBF and MTT between these bolus tracking techniques.

**Supplementary Information:**

The online version contains supplementary material available at 10.1007/s10334-022-01038-y.

## Introduction

Gaining insights into the perfusion of brain parenchyma is important for a large number of neurological conditions spanning from acute stroke and trauma to cancer and neurodegenerative diseases [1–3]. This has made perfusion imaging part of the daily diagnostic practice within the field of neuroradiology, typically performed using bolus tracking techniques [4, 5].

The basis for bolus tracking-based perfusion imaging is the injection of a contrast agent into a vein and the recording of its influence on the measured signal as it passes through a tissue of interest. Insights about how the blood passes through tissue, i.e., perfusion can, according to indicator-dilution theory, be obtained by the use of an indicator [6, 7]. The contrast agent can be regarded an indicator if its flow through tissue is similar to that of blood, if it is detectable, and if it does not in other ways disturb the system that it is used to probe [7]. Through analysis of the indicator concentration–time curve together with insights in how the delivery of the indicator to the tissue took place, parameters that characterize the perfusion can be derived. Frequently used perfusion parameters include the cerebral blood volume (CBV), the cerebral blood flow (CBF), and the mean transit time (MTT) [8].

Two clinically available techniques frequently used for cerebral perfusion imaging are dynamic computed tomography perfusion (CTP) and dynamic susceptibility contrast MRI (DSC-MRI) [9], both enabling estimation of the aforementioned perfusion parameters. DSC-MRI has the advantage of providing perfusion imaging without the ionizing radiation associated with CTP. Furthermore, MR-based perfusion images are typically acquired together with several other MR images with different contrasts, providing a comprehensive basis for radiological evaluation. On the other hand, MRI is still not as readily available as CT, and in the acute setting, CT scanning is preferred to avoid the risk of bringing in non-responsive patients with unknown medical implants into the magnetic fields of an MRI scanner [3].

Even though CTP and DSC-MRI are both based on the same indicator-dilution theory and are intended to probe the same perfusion characteristics, some discrepancies have been observed regarding CBF and MTT in direct comparison between the two techniques [10]. This is reasonably explained by the different imaging setups for CTP and DSC-MRI, including for example different injection durations, noise levels, and temporal resolutions [3, 8]. Since the injection duration, which is approximately four times longer for CTP compared with DSC-MRI, has a very strong influence on the appearance of the measured signal–time curves, we hypothesize that differences in CBF and MTT estimates between CTP and DSC-MRI to some degree can be attributed to the duration of the bolus injection.

In theory, the derivation of perfusion parameters benefits from keeping the injection duration as short as possible. In practice, however, the injection speed must be limited due to the risk of rupturing the vein. For this reason, it is set slightly slower for CTP due to the higher viscosity of the iodine-based contrast agent. The duration of the contrast agent injection is dictated not only by the speed of injection, but also by the amount of contrast agent to be injected. In CTP, a larger volume of contrast agent must be injected to achieve a sufficient signal effect. This is the primary cause for the longer injection when compared to DSC-MRI.

In a study where patients underwent CTP and DSC-MRI examinations, a direct comparison showed lower CBF values for CTP than for DSC-MRI [10]. The discrepancy was mainly seen for high CBF levels, meaning that it is likely to impact also relative measures as the reference region usually is placed in white matter which has a comparatively low CBF. This difference in estimated CBF values between CTP and DSC-MRI is an obvious problem when comparing results based on the two modalities from different studies, but can also have an actual impact on clinical decision-making. This may, for example, occur within tumour diagnostics, if a follow-up examination needs to be moved from DSC-MRI to CTP due to a newly implanted pacemaker.

The literature on the topic of bolus injection durations for perfusion imaging is not in perfect agreement. In two previous publications, it was stated that lower injection rates would not impair the accuracy of estimates [4, 11] as bolus dispersion due to long-injection durations are accounted for in the deconvolution process. On the other hand, in repeated DSC-MRI bolus tracking experiments using different contrast agents, parameter estimates showed only weak correlations [12]. Another study that evaluated CTP injection schemes with durations as long as 18 s deemed estimates of CBF from low injection rate imaging “more or less reliable” [11]. In a study by van Osch et al., the effects of bolus injection duration and its negative impact on perfusion parameter estimates in DSC-MRI were studied [13]. However, to our knowledge, there have been no direct comparisons of the effect of bolus (injection) duration in combination with typical noise levels of CTP and DSC-MRI in clinically relevant perfusion scenarios.

The aim of this study was to conclude whether the discrepancies regarding cerebral blood flow (CBF) and mean transit time (MTT) between dynamic computed tomography perfusion (CTP) and dynamic susceptibility contrast MRI (DSC-MRI) can be attributed to the different injection durations, noise levels of the imaging techniques, or a combination of these two.

## Materials and methods

The general methodological approach to address the aim of this study was via simulations to explore the effect of injection duration on a clinically relevant range of perfusion scenarios. Extra care was given to the separation of noise effects from injection duration effects and to minimize effects from the parameter estimation by selecting specifically optimized regularization. The simulation model was designed to reproduce in vivo data from gray and white matter in healthy volunteers.

### Study subjects

Five elderly, healthy individuals (68–75 years, 4 males) were scanned using DSC-MRI and CTP on 2 consecutive days, at the same time of day to avoid diurnal fluctuations. The data have been included in previous studies addressing absolute perfusion quantification in DSC-MRI and CTP [10], and in a comparison of perfusion in gray and white matter regions between patients with idiopathic normal pressure hydrocephalus and age-matched, healthy individuals [14, 15]. All subjects gave their informed consent for inclusion prior to participating in the study. The study was conducted in accordance with the Declaration of Helsinki and was approved by the regional ethical review board in Gothenburg (Dnr154-05).

### CT imaging

CT data were acquired using a General Electric, Lightspeed PRO 16, with a 16-channel multi-detector array. The examination covered four adjacent 5-mm sections immediately above the posterior commissure, parallel to a line between the posterior commissure and the root of the nose. Special care was taken to ensure that a similar slice angulation between CT and MR imaging was achieved. The acquisition parameters were 80 kV, 200 mA, 99 repetitions, four images per cine scan, scan repetition time: 0.5 s, acquisition time: 49.5 s, FOV: 250 × 250 mm^2^, and matrix: 512 × 512. Five seconds after the CT scanning was started, a bolus (4 ml/s) of 50 ml Iopromide (300 mg iodine/ mL, viscosity 4.7 mPa·s @ 37 °C, Ultravist, Schering, Berlin, Germany), followed by a saline flush, was injected into the right ante-cubital vein. Injection duration was 12.5 s.

### MR imaging

MR data were acquired with a 1.5T Philips Gyroscan Intera 9.1 system (Philips Medical Systems, Best, The Netherlands) using a six-channel SENSE head coil. For anatomical reference, a transverse fluid-attenuated inversion-recovery (FLAIR) image was acquired with echo time (TE): 100 ms, repetition time (TR): 9000 ms, inversion time (TI): 2500 ms, slice thickness: 3 mm, 44 slices, field of view (FOV): 230 × 230 mm^2^, image acquisition matrix: 192 × 192 reconstructed to 256 × 256. DSC-MR images were acquired using a transverse segmented gradient-echo echo-planar imaging (EPI) sequence with two segments, TR: 500 ms, resulting in a scan repetition time of 1000 ms, flip angle: 40°, TE: 30 ms, slice thickness: 5 mm, 12 slices, acquisition time: 100 s, FOV: 230 × 230 mm^2^, image acquisition matrix 128 × 79 reconstructed to 128 × 128. Ten seconds after the DSC acquisition was started, a bolus (5 ml/s) of 0.1 mmol/kg body weight Gd-DTPA (0.5 mmol/ml Magnevist, viscosity 2.9 mPa·s @ 37 °C, Schering, Berlin, Germany), followed by a saline flush, was injected into the same ante-cubital vein as during the CTP examination. Injection duration was approximately 3 s.

### In vivo data extraction

**Fig. 1:**
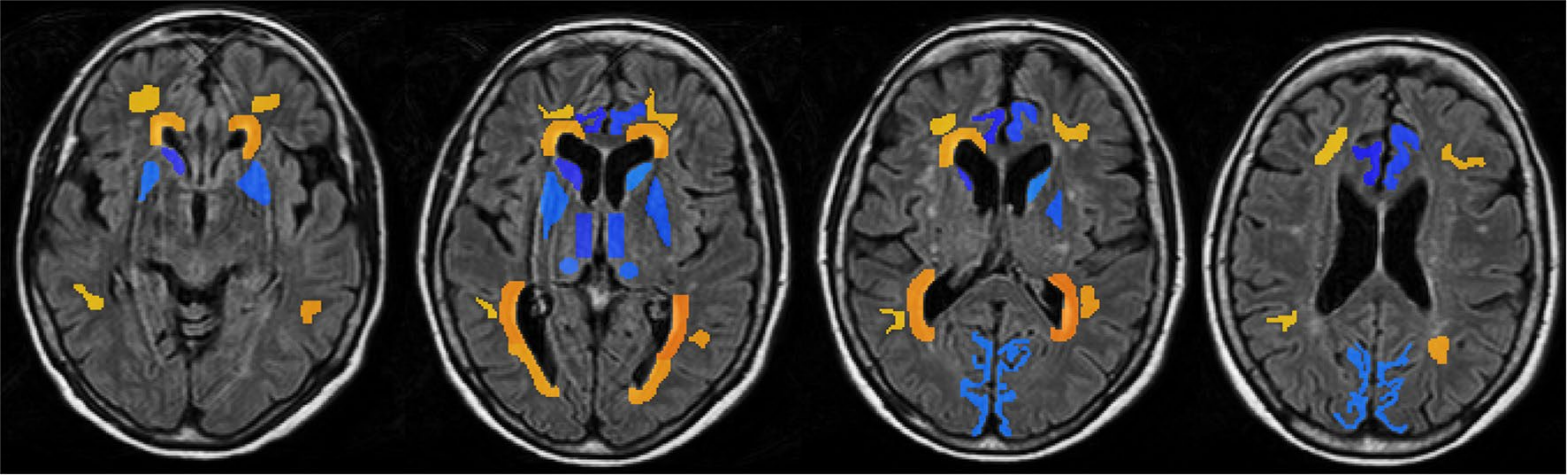
A subset of four slices of FLAIR MRI data, showing manually delineated ROIs with white matter in nuances of orange (normal appearing, anterior periventricular, and posterior periventricular) and gray matter in nuances of blue (cingulate gyrus, caudate head, lentiform nucleus, central thalamus, periventricular thalamus, and occipital cortex). A more detailed description of delineated ROIs has been published previously [14, 15]

Regions of interest (ROIs) covering gray and white matter regions were manually delineated on the FLAIR dataset by an experienced neuroradiologist; see Fig. 1. The ROIs were transferred to the perfusion data sets via registration of the FLAIR dataset to the DSC-MRI and CTP data, respectively. Both 4D data volumes were averaged over the temporal dimension to generate 3D volumes for the registration. For DSC-MRI, registration was performed using RegAladin, which is part of the NiftyReg software package [16]. Due to the limited anatomical coverage of four slices for the CTP data, rigid registration was performed manually using the software 3D slicer [17] (ver. 4.3, http://www.slicer.org).

In vivo signal–time curves were obtained by averaging ROI voxels for each time point. To suppress periodic signal variations related to arterial-inflow effects and pulsation, DSC-MRI concentration–time curves were filtered by removing frequency components above a manually chosen threshold in the Fourier domain. The DSC-MRI data were temporally registered to the first volume using the software package SPM (Wellcome Trust Centre for Neuroimaging, UCL, UK), while the CTP data, consisting of only four slices, were not registered along the temporal domain.

For both CTP and DSC-MRI, the arterial input function (AIF) was obtained semi-automatically from the left hemisphere middle cerebral artery. Voxels that ranked among the 20% with largest peak height, early arrival time, and of similar shape to the mean tissue concentration time curve were automatically preselected. Among these voxels, the one that showed the sharpest peak and without signs of signal saturation was manually selected [14].

### Simulations

**Fig. 2:**
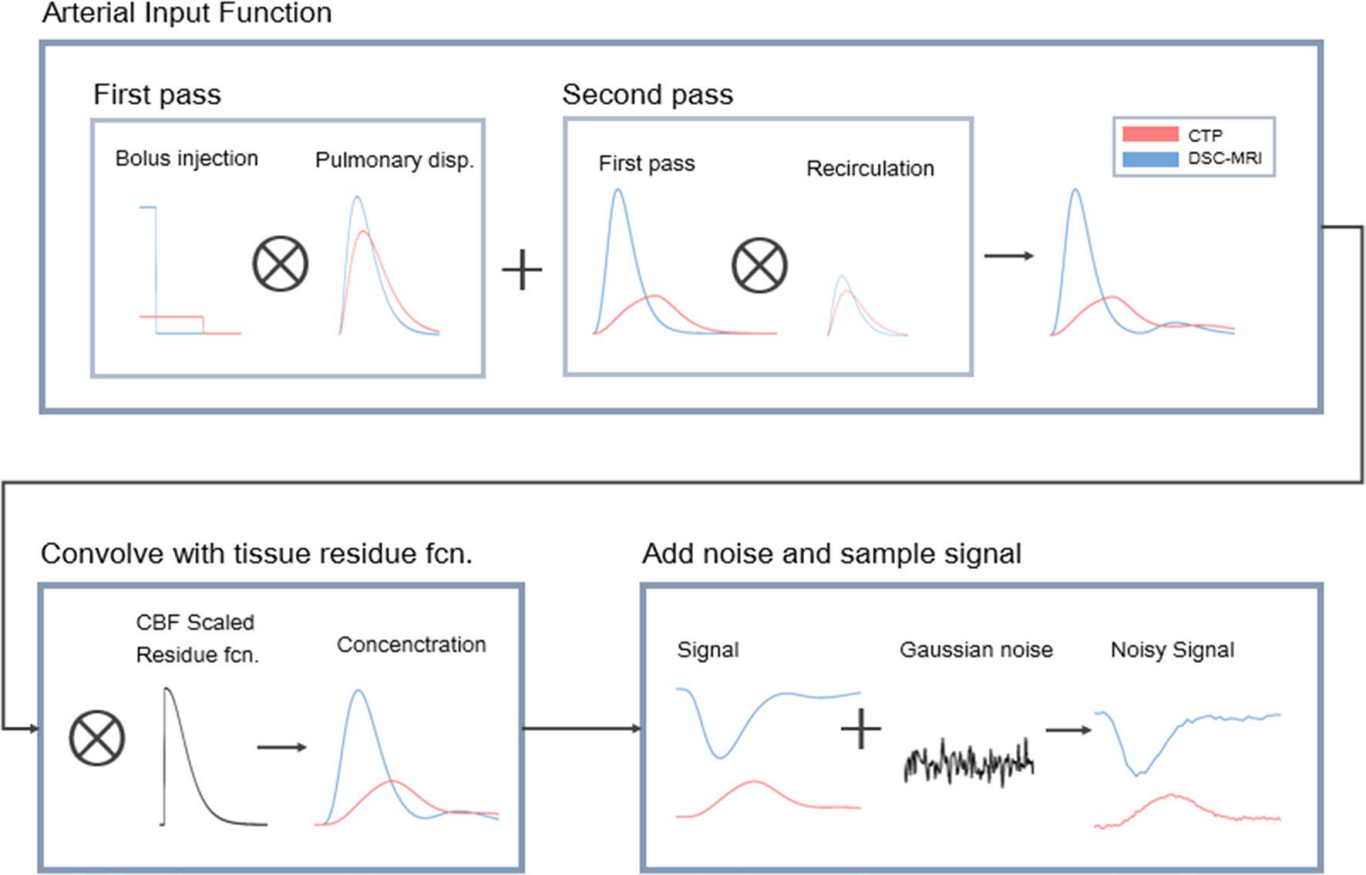
Overview of the simulation of noisy signal–time curves for CTP (red) and DSC-MRI (blue)

**Fig. 3:**
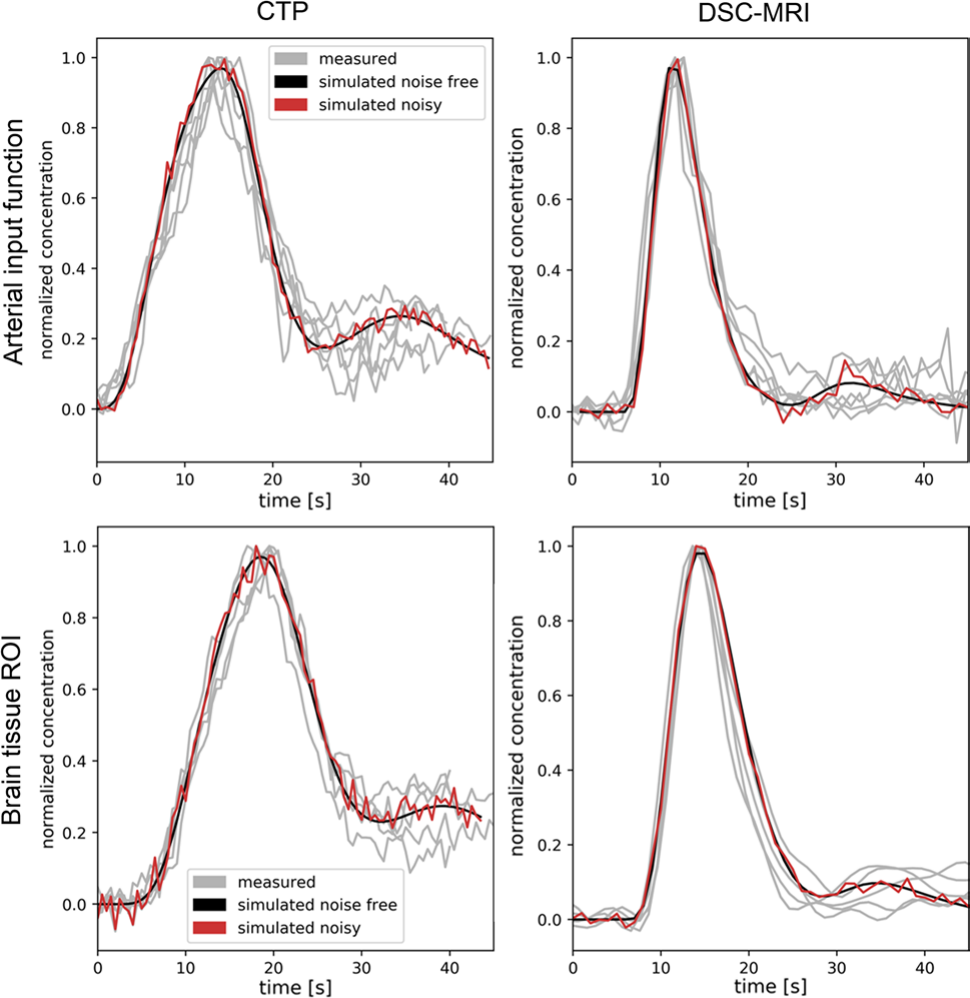
Measured, simulated noisy and simulated noise-free concentration–time curves. The noisy time curves were normalized to peak amplitude and the noise-free time curves were empirically scaled to mean peak amplitude of the noisy curves. The measured data (gray lines) were taken from ROIs that covered the right nucleus lentiformis (gray matter) for each subject

**Fig. 4:**
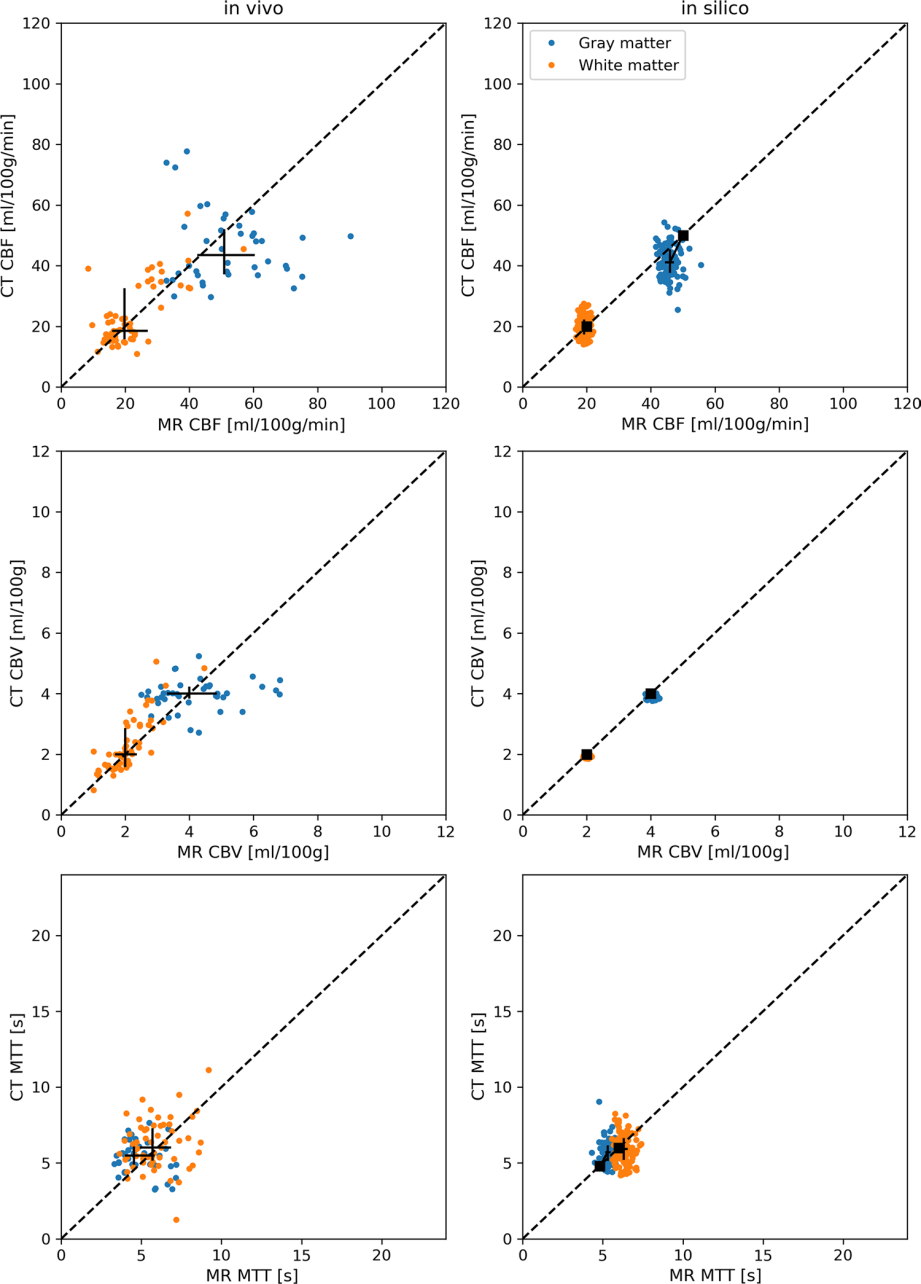
Scatter plots produced using oSVD with regularization selected based on proposed values of Wu et al. [22]: OI = 0.065 for DSC-MRI and OI = 0.035 for CTP based on their respective CNR levels. Simulated data (right hand column of scatter plots) have ${\mathrm{CNR}}_{\mathrm{AIF}} \mathrm{=45,} ~{\mathrm{CNR}}_{\mathrm{tissue}}=\mathrm{90~and~30}$ for DSC-MRI and CTP respectively, corresponding to the approximate CNR of measured data (left-hand column of scatter plots). The square symbol represents the true values, the center of the cross is the median, and the axes of the cross are the inter-quartile range of estimates for each tissue group. A dashed unity line is provided to aid assessment of relative bias between CTP and DSC-MRI

**Fig. 5:**
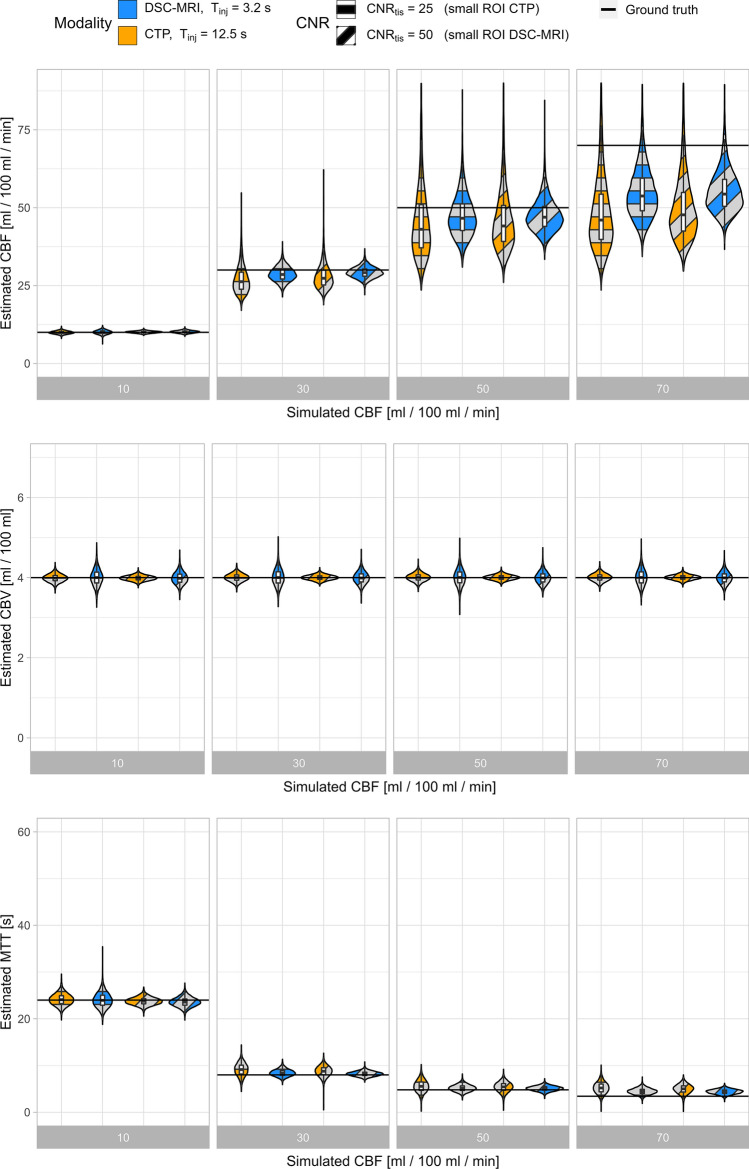
Perfusion estimates from simulations of small ROI analysis in CTP with ${T}_{\mathrm{inj}}$= 12.5 s (orange) and DSC-MRI with ${T}_{\mathrm{inj}}$= 3.2 s (blue), with ${\mathrm{CNR}}_{\mathrm{tiss}}=$ 25, 50 (orthogonal and diagonal pattern) in CTP and DSC-MRI, respectively. Combinations of the two levels of CNR and ${T}_{\mathrm{inj}}$ were evaluated yielding a total of four estimates per simulated perfusion level. The violin plots include the kernel density profile (violin sides) as well as a boxplot showing the median and inter-quartile range. Estimates of CBV, CBF, and MTT estimated using oSVD with regularization selected individually based on the lowest resulting MAE for each simulated perfusion scenario, CBV = 4/100 ml and ${\mathrm{CNR}}_{\mathrm{AIF}}=$ 45

**Fig. 6:**
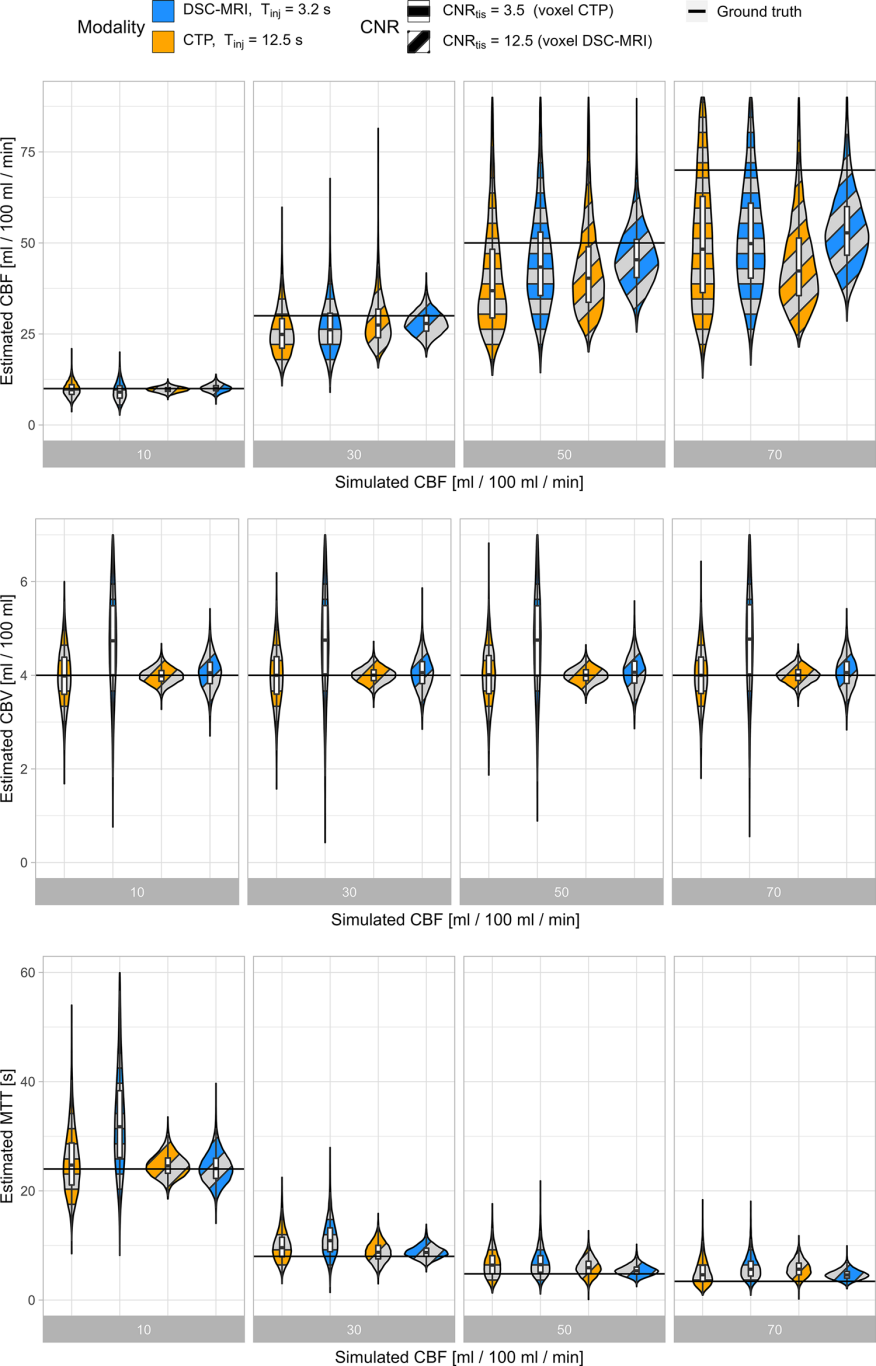
Perfusion estimates from simulations of voxel-wise analysis in CTP with ${T}_{\mathrm{inj}}$= 12.5 s (orange) and DSC-MRI with ${T}_{\mathrm{inj}}$= 3.2 s (blue), with ${\mathrm{CNR}}_{\mathrm{tiss}}=$ 3.5, 12.5 (orthogonal and diagonal pattern) in CTP and DSC-MRI, respectively. Combinations of the two levels of CNR and ${T}_{\mathrm{inj}}$ were evaluated yielding a total of four estimates per simulated perfusion level. The violin plots include the kernel density profile (violin sides) as well as a boxplot showing the median and inter-quartile range. Estimates of CBV, CBF, and MTT estimated using oSVD with regularization selected individually based on the lowest resulting MAE for each simulated perfusion scenario, CBV = 4/100 ml and ${\mathrm{CNR}}_{\mathrm{AIF}}=$ 45

Simulation of signal–time curves was performed according to Fig. 2; each step covered in detail in this section. Parametric models of the AIF and the tissue residue function were used to simulate tissue concentration–time curves. Two sets of data were produced. In the first set, the parameters of the AIF and tissue models were set to reproduce the in vivo data, including the noise levels and temporal resolutions. The resulting time curves are shown together with in vivo data in Fig. 3. This dataset was finally used to reproduce the characteristics of the resulting perfusion parameter estimates from CTP and DSC-MRI, respectively, as seen in Fig. 4. With the AIF and tissue model parameters established, the second dataset was created. In these simulations, the effect of injection duration was evaluated on a larger range of perfusion scenarios and noise levels, and with a common temporal resolution of 1 s for both DSC-MRI and CTP time curves. This dataset was the basis for the perfusion estimates in Figs. 5, 6 and Sup. Figs. S2–S7.

To limit numerical errors related to convolutions, the noise-free time curves were simulated using a time vector with an equidistant sample grid with 0.01 s time steps. Time curves were finally subsampled according to relevant scan repetition rates. Dependencies in perfusion estimates due to deconvolution aspects were minimized using individually selected levels of deconvolution regularization for each perfusion scenario. Table 1 summarizes the noise levels and perfusion scenarios used in this study.

**Table 1:** Summary of simulations included in figures, including simulated perfusion parameters CBV and CBF as well as tissue concentration CNRs

Fig. and Sup. Fig	CBV [ml/100 ml]	CBF [ml/100 ml/min]	${\mathrm{CNR}}_{\mathrm{tis}}$ DSC-MRI [a.u.]	${\mathrm{CNR}}_{\mathrm{tis}}$ CTP [a.u.]
3, 4	4	50	90	30
4	2	20	90	30
5, S6, S7	4	10, 30, 50, 70	25, 50	25, 50
6	4	10, 30, 50, 70	3.5, 12.5	3.5, 12.5
S2	4	10, 30, 50, 70	50, 100	50, 100
S3	2	5, 15, 25, 35	25, 50	25, 50
S4	2	5, 15, 25, 35	50, 100	50, 100
S5	2	5, 15, 25, 35	3.5, 12.5	3.5, 12.5

Parameters either set constant for all simulations or derived from parameters listed in the table include ${CNR}_{AIF}$= 45, residue function shape parameter $\alpha$ = 3, MTT = CBV/CBF, and CTH = MTT/$\sqrt{\alpha }$

For the results shown in Figs. 5, 6 and Sup. Figs. S1–S7, time curves of two different modalities (including injection durations), five different noise levels, and eight perfusion levels were simulated. For each scenario, 10 000 noise instantiations were produced, yielding a total of 800 000 individual time curves that were analyzed.

### Arterial input function

The first AIF bolus passage was simulated as the convolution between the bolus injection time curve and a dispersion kernel representing the transit time distribution through the pulmonary circulation [18]. Bolus injection was modelled as a box car function with length corresponding to injection duration and height corresponding to contrast agent concentration

1 $$B\left(t\right) =\left\{\begin{array}{ll} A& a<t<b\\ 0& t<a ~ \mathrm{or} ~b<t,\end{array}\right.$$

where *A* is the amplitude between time points $a$ and $b$. The injection durations were set to 12.5 and 3.2 s for CTP and DSC-MRI, respectively. For CTP, the bolus duration was given by the power injector speed and fixed volume of contrast agent, while for DSC-MRI, the duration corresponded to what would be used for a patient with weight of 80 kg.

The dispersion kernel for the pulmonary circulation was described by a gamma variate function

2 $$\varphi \left(t;\mathrm{\alpha },\upbeta \right)=\frac{1}{{\beta }^{\alpha }\Gamma (\alpha )}{t}^{\alpha -1}{\mathrm{e}}^{-\frac{t}{\beta }},$$

with parameters $\mathrm{\alpha }$ and $\beta$.

Recirculation was modelled by adding a delayed, dampened, and dispersed replicate, giving the following expression for the AIF:

3 $${C}_{\mathrm{a}}\left(\mathrm{t}\right)=\left(B\left(t\right)+{\mathrm{e}}^{-\uplambda \delta }B\left(t-\delta \right)\otimes \varphi \left(t;\mathrm{\alpha },\upbeta \right)\right)\otimes \varphi \left(t;\mathrm{\alpha },\upbeta \right),$$

where $\delta$ is a delay between recirculations, $\lambda$ is an exponential dampening factor, and $\otimes$ denotes convolution.

Dispersion and recirculation parameters were selected empirically to produce concentration–time curves resembling the in vivo data. For CTP, dispersion parameters were set to ${\mathrm{\alpha }}_{\mathrm{disp}}=3$ and ${\upbeta }_{\mathrm{disp}}=2.4$, while recirculation parameters were set to $\uplambda =0.11$ s^−1^ and $\delta =13$ s. For DSC-MRI dispersion, parameters were set to ${\mathrm{\alpha }}_{\mathrm{disp}}=3$ and ${\upbeta }_{\mathrm{disp}}=1.8$, while recirculation parameters were set to $\uplambda =0.13$ s^−1^ and $\delta =16$ s.

### Tissue response function

The flow scaled tissue residue function was obtained by the following expression:

4 $${R}_{\mathrm{CBF}}\left(\mathrm{t}\right)=\mathrm{CBF}\cdot R\left(t\right)=\mathrm{CBF}\cdot {\int }_{\mathrm{t }}^{\infty }h\left(\uptau \right)\mathrm{d\tau },$$

where $h\left(\uptau \right)$ denotes the distribution of transit times. $h\left(\tau \right)$ was assumed to be given by a gamma variate distribution [19, 20] (same as Eq. 2), with a fixed ${\alpha }_{\mathrm{res}}=3$ and where ${\beta }_{\mathrm{res}}=\mathrm{MTT}/{\alpha }_{\mathrm{res}}$ varied with the simulated perfusion scenario according to the gamma variate distributions closed form expression for MTT. Simulated standard deviation of transit times (capillary transit time heterogeneity; CTH) was thus a dependant of MTT according to $\mathrm{CTH}=\mathrm{MTT}/\sqrt{{\alpha }_{\mathrm{res}}}$ [21].

### Signal–time curves

Tissue concentration–time curves were given by

5 $${C}_{t}\left(t\right)={C}_{\mathrm{a}}\left(t\right)\otimes {R}_{\mathrm{CBF}}\left(t\right)$$

and transformed to signal–time curves following the signal models for the respective modality:

6 $$S\left(t\right)=\left\{\begin{array}{cc}{S}_{0}{\mathrm{e}}^{-K\cdot \mathrm{TE}\cdot C(t)}& \mathrm{DSC-MRI}\\ {S}_{0}+\kappa \cdot C\left(t\right)& \mathrm{CTP}\end{array},\right.$$

where ${S}_{0}$ is the baseline signal intensity before the contrast agent enters the tissue, $\mathrm{TE}$ is the echo time, and $K$ is the constant relating the concentration to the susceptibility effect on relaxivity, here approximated to be linear. $\kappa$ is the linearity constant relating the concentration of contrast agent to Hounsfield units. Note that, herein, the linearity constants $K$ and $\kappa$ include the correction factor for haematocrit as well as the brain tissue density. Baseline signal levels for tissue curves and AIFs were set at 100 and 30 HU for DSC-MRI and CTP, respectively. The peak signal deviation from baseline, at concentration maximum in a reference tissue with a CBF of 60 ml/100 ml/min, was set to −40 for DSC-MRI and 20 HU for CTP. For the AIF, the maximum signal difference was set at −60 and 100 HU for DSC-MRI and CTP, respectively.

### Noise

The contrast-to-noise ratio (CNR) was defined in the concentration domain as follows:

7 $$\mathrm{CNR}=\frac{{C}_{\mathrm{max}}}{{\sigma }_{\mathrm{baseline}}},$$

${C}_{\mathrm{max}}$ is the peak concentration and ${\sigma }_{\mathrm{baseline}}$ is the standard deviation of the concentration–time curve during baseline [13].

AIF, ROI, and voxel CNR representative for the in vivo data were approximately 45, 90, and 12.5 for DSC-MRI, and 45, 30, and 3.5 for CTP, respectively. To account for varying ROI sizes in measured data, the simulations included a range of CNRs, 12.5, 50, 100 for DSC-MRI and 3.5, 25, and 50 For CTP. Simulated noise levels and perfusion scenarios are summarized in Table 1. Each CNR was defined for a reference concentration–time curve with a CBF of 60 ml/100 ml/min. The noise amplitude that achieved this CNR was then applied for each perfusion level in this data subset. Effectively resulting in a lower CNR for perfusion scenarios with a CBF below the reference value. This resembles the varying CNR levels found in an in vivo perfusion dataset, resulting from the static noise properties of the imaging modality.

### Estimation of perfusion parameters

Deconvolution was performed using the oscillatory index Singular Value Decomposition (oSVD) method, which is based on a circular convolutional matrix, providing a delay insensitive estimate of the tissue response function, ${\widehat{R}}_{\mathrm{CBF}}\left(t\right)$ [22]. Perfusion parameters were then derived from ${\widehat{R}}_{\mathrm{CBF}}\left(t\right)$ using

8 $$\begin{array}{c}\begin{array}{c}\mathrm{CBF}=\begin{array}{c}\mathrm{max}\\ i\in n\end{array}{\widehat{{\text{R}}}}_{\mathrm{CBF}}\left(i\right)\\ \end{array}\\ \mathrm{CBV}=\Delta t\cdot {\sum }_{i\in n}{\widehat{\mathrm{{\text{R}}}}}_{\mathrm{CBF}}\left(i\right)\\ \begin{array}{c}\\ \mathrm{MTT}=\frac{\mathrm{CBV}}{\mathrm{CBF}},\end{array}\end{array}$$

where $n=\{1,\dots ,N\}$ denotes the sample indices.

To present parameter estimates on a relevant scale, the factors $K$ and $\kappa$ of Eq. 6 were applied to estimates of CBF and CBV. The in vivo CBV and CBF estimates were re-scaled to enable evaluation of estimation bias in in vivo data and comparisons with simulated data. Scaling factors were chosen, such that the median ROI-wise CBV estimate from each patient and tissue type equalled the CBV in the corresponding simulations, i.e., 2/100 ml for white matter and 4/100 ml for gray matter. In this way, the inter-individual variation (including, e.g., partial volume effects) was eliminated, but intra-individual and inter-ROI variation was preserved.

### Levels of oSVD regularization

It has been shown that oSVD-based deconvolution methods tend to underestimate high CBF [23, 24] and provide non-physiologically oscillating residue functions [23]. To focus on the effect of injection duration and noise, and minimize the effects of a suboptimal regularization, deconvolution was performed with regularization that was optimized for each simulated perfusion scenario (set of CBF, CBV and CTH). The optimal regularization was defined as the oscillatory index, which from a set of 40 logarithmically spaced regularization levels between 10^-3 and 10^0.5, produced the lowest CBF mean absolute error (MAE) based on 10 000 Monte Carlo noise iterations (see Sup. Fig. S1 and Sup. Table S1).

### Statistical analysis

The agreement of resulting parameter estimates with ground-truth levels was quantified by the percentage of parameter estimates below the ground-truth value. Agreement among distributions of parameter estimates obtained from different simulation scenarios relevant to compare was quantified by the overlap of the distributions as described previously [25, 26].

## Results

### Gray and white matter perfusion in vivo and in silico

The shape of in vivo concentration–time curves was to a high degree replicated via simulations, as shown in Fig. 3. Also, while showing some differences in variability between simulations and in vivo data in Fig. 4, the simulations reproduced the general characteristics of perfusion estimates in the in vivo data. While both CTP and DSC-MRI underestimated CBF in the higher perfused gray matter tissue, the negative bias was larger for CTP, see the top row of Fig. 4, where CTP estimates to a larger extent were found below the unity line.

### The effects of CNR and injection duration in simulations

This study includes simulations spanning a range of perfusion scenarios, CNRs, and injection durations for the estimation of the perfusion parameters CBF, CBV and MTT. Herein, we focus on how these factors impacted on bias and variability of the perfusion estimates.

As expected and in accordance with previous simulation studies using oSVD-based perfusion estimation [22, 24, 27], there was a trend towards an increased negative bias and variability for CBF estimates with increasing CBF. This property of the deconvolution technique relates to its limitations in recovering high-frequency components in the residue function regardless of CNR level. As seen in Figs. 5 and 6, Sup. Figs. S2–S7 and Sup. Tables S2–S9, the negative bias in CBF estimates was larger and increased more with increasing CBF for the longer injection duration typical of CTP when compared to that of DSC-MRI. When comparing results pairwise between DSC-MRI and CTP for a given CBF, the different injection durations proved to be a larger source of bias and variability than the different noise levels for ROI-wise analyses, as seen for example in the upper right of Sup. Figs. S6 and S7. In fact, when isolating the injection duration effects for each modality as shown in Sup. Figs. S6 and S7, the difference between a 3.2 and 12.5 s injection duration can explain as much as 16 and 31% of the bias at CNR 50 and for CBF 70 ml/100 g/min for DSC (Sup. Fig. S6) and CT perfusion (Sup. Fig. S7), respectively. Comparing CBF estimates between two ROI CNR levels for the same imaging modality, as found in Fig. 5 and Sup. Figs. S2–S4, S6–S7 and Sup. Tables S2, S4–S6, S8–S9, it seems that when oSVD regularization is chosen individually for each scenario, a decreased noise amplitude did not lead to very large improvements in the bias and variability of the resulting estimates.

There was no apparent relationship found between neither bias nor variability of CBV and the simulated CBF level. This was seen for all simulated CNR levels and for both DSC-MRI and CTP in Figs. 5 and 6, Sup. Figs. S2–S5 and Sup. Tables S2–S7. When comparing the effect that injection duration and noise had on resulting parameter estimates, the effects on CBV estimates showed to be smaller in terms of bias and variability than what was found for CBF and MTT. CBV variability increased with decreasing CNR for both CTP and DSC-MRI, but slightly more so for DSC-MRI as can be seen for example in the middle row of Fig. 5. CBV variability was larger for the shorter injection duration found in DSC-MRI, and this was also consistent over simulated CNR and CBF. For a CNR level corresponding to voxel-wise analyses, a larger positive bias was seen for the CBV estimate for the low CNR = 3.5 level, and this effect can be seen in the second violin for all CBF levels of Fig. 6 and Sup. Fig. S5 and in Sup. Tables S3 and S7.

The simulations in Figs. 5, 6 and Sup. Figs. S2–S5 not only include the effect of the injection duration, but also the specific signal models of each simulated imaging modality (orange/blue violins). To illustrate the effect of each signal model in relation to the effect of the injection duration, simulations covering both injection durations individually for each modality were produced. The results, found in Sup. Figs. S6 and S7 and Sup. Tables S8 and S9, show that primary cause of estimate bias when comparing the two modalities in Figs. 5 and 6 as well as in Sup. Figs. S2–S5 is indeed the injection duration effect.

As a result of the generally small bias for CBV and the central volume theorem in Eq. (8), MTT estimates showed similar, but inverse trends compared to those seen for CBF. Especially for the low CNR simulations found in Fig. 6 and Sup. Fig. S5, MTT shows overestimations for both DSC-MRI and CTP. However, for CNR levels 25, 50 and 100 typical of large and small ROI analyses seen in Fig. 5, Sup. Figs. S2–S4 and Sup. Tables S4–S7, the bias in MTT estimates from DSC-MRI was smaller than those from CTP.

## Discussion

The results of this study show that CTP and DSC-MRI with oSVD-based deconvolution underestimate high levels of CBF. However, the bias and variability of CBF estimates were larger for CTP than for DSC-MRI. We have shown that the different injection durations could explain these differences to a large extent, while the two modalities different noise levels and signal models could only account for a small portion of the differences in parameter estimates.

Quantitative perfusion imaging is associated with a number of challenges [28, 29]. For example, for DSC-MRI, the relation between tracer concentration and relaxation rate is not the same between arteries and capillaries. Furthermore, the AIF can be subject to partial volume and phase effects, pixel shift effects, and signal saturation [30]. The AIF in CT perfusion can also be subject to partial volume effects, even though the pixel resolution is typically higher for CTP compared to DSC-MRI for clinical acquisitions. While multiple factors differ between DSC-MRI and CTP, our results show that the effect caused by the length of the injection duration can explain a substantial part of the differences between perfusion parameter estimates of CBF and MTT.

Comparing the effect of injection duration on parameter estimates, with all other factors held constant (as in Sup. Figs. S6–S7 and Sup. Tables S8 and S9) showed that an injection duration of 12.5 s (typical for CTP) yielded an increased bias for both CBF and MTT estimates when compared to 3.2 s injections (typical for DSC-MRI). Deconvolution in bolus tracking imaging is typically considered an ill conditioned (and ill posed) inverse problem [8]. That is, small differences in data can have a large impact on estimates. As the injection duration increases, the same tissue response is encoded into a temporally longer curve. Based on our results, we reason that such an encoding may affect the condition number of the inverse problem, causing a more challenging deconvolution. On the other hand, CBV estimates can be obtained without deconvolution [31], and if noise is normally distributed, may even benefit from longer injection durations as more datapoints contribute to the estimation. While CBV estimates generally were of high accuracy and precision, ever so slightly lower variabilities in CBV estimates from simulations with 12.5 s injection durations (typical for CTP) support this reasoning.

The primary effect of an increased noise level was an increase in variability and a slight increase in negative bias of CBF estimates. When considering the highest noise levels simulated, typical for voxel-based analysis, a clearer increase in CBF estimate bias was found for both modalities. Evaluating estimates from DSC-MRI at the lowest noise level typical for CTP examinations yielded a substantial increase in CBV estimate bias (leftmost violin for each CBF in Fig. 6). A focused analysis showed that this was caused by the combination of an increased noise level and the log-transform in the signal to concentration transformation of Eq. (6) for DSC-MRI (data not shown). At low CNR, the noise distribution in the DSC-MRI signal domain is no longer zero-mean, resulting in such biased CBV estimates. This evaluation was made strictly for comparative purposes and in practice a voxel-based analysis with DSC-MRI will typically produce time curves with a higher CNR.

Differently biased perfusion estimates in DSC-MRI and CTP could have implications in the clinic. For example, if a change of modality from DSC-MRI to CTP is made during the cause of a cancer treatment, an underestimation of CBF in highly perfused areas in CTP could potentially be misinterpreted as a response to treatment. However, current standard procedure includes the reviewing of CBV maps, which we have shown to be relatively insensitive to bolus injection duration, noise, and the signal model. We can conclude that this seems to be the most robust estimate for when a change of modality is a must. If perfusion estimates CBF and MTT from these modalities are to be compared, whether it be in a clinical or a research setting, awareness of the injection duration effect and when it comes into play may be critical for correct interpretations.

This study is associated with some limitations. First, while SVD-based deconvolution techniques are frequently used in perfusion imaging, several other methods could have been used in this study. While the effect of this limitation was minimized using optimally selected oscillatory indexes, this could be applied only in the simulation-based experiments. Also, we used a linear model for the relaxivity dependance on contrast agent concentration in DSC-MRI. However, this relationship has been shown to be somewhat non-linear [32] and the use of a linear model may have contributed a negative bias to the CBF estimates in the experimental evaluation shown in Fig. 4. As for the simulations, the linear model was used both when converting concentration to signal time curves, and, in the conversion back to the concentration domain during analysis. Therefore, this effect will to a large extent have canceled out. Another limitation was that the comparison of estimates from simulated CTP and DSC-MRI time curves was limited to a temporal resolution set to 1 s for both modalities. Increasing the temporal resolution may to some extent increase the accuracy of resulting parameter estimates. For MRI, this is currently challenging to achieve with whole brain coverage, and for CTP, the benefit is not obvious as the smoother curve shape of CTP can be accurately captured also at the current temporal resolution.

## Conclusion

The results of this study suggest that differences in estimates of cerebral blood flow (CBF) and mean transit time (MTT) seen in comparisons between CTP and DSC-MRI to a large degree can be attributed to the different injection durations and to some degree to the different noise levels. More specifically, by the use of simulations, we have shown that at a given noise level, the longer injection duration typical for CTP will produce CBF and MTT estimates with increased bias and variability when compared to that typical of DSC-MRI. These aspects of bolus tracking perfusion are important to be aware of when comparing results from research studies, in the clinic or when designing data acquisition protocols.

## Supplementary Information

Below is the link to the electronic supplementary material.

Supplementary file1 (PDF 1417 KB)
