# Supplementary material for: Effects of bolus injection duration on perfusion estimates in dynamic CT and dynamic susceptibility contrast MRI
Source: MAGMA. 2022 Sep 17;36(1):95–106. doi: 10.1007/s10334-022-01038-y (PMC9992234; doi:10.1007/s10334-022-01038-y)
Supplement: Supplementary file 1 — Supplementary file1 (PDF 1417 KB) [file 10334_2022_1038_MOESM1_ESM.pdf]

Finding the optimal regularization for the deconvolution

To separate the effect of bolus injection duration from the effects of a suboptimal regularization, circular SVD deconvolution was performed with the oscillatory index regularization optimized for each perfusion scenario. The optimal regularization was defined as the oscillatory index, which from a set of 40 logarithmically spaced regularization levels between 10^-3 and 10^0.5, produced the lowest CBF MAE based on 10 000 Monte Carlo noise iterations.

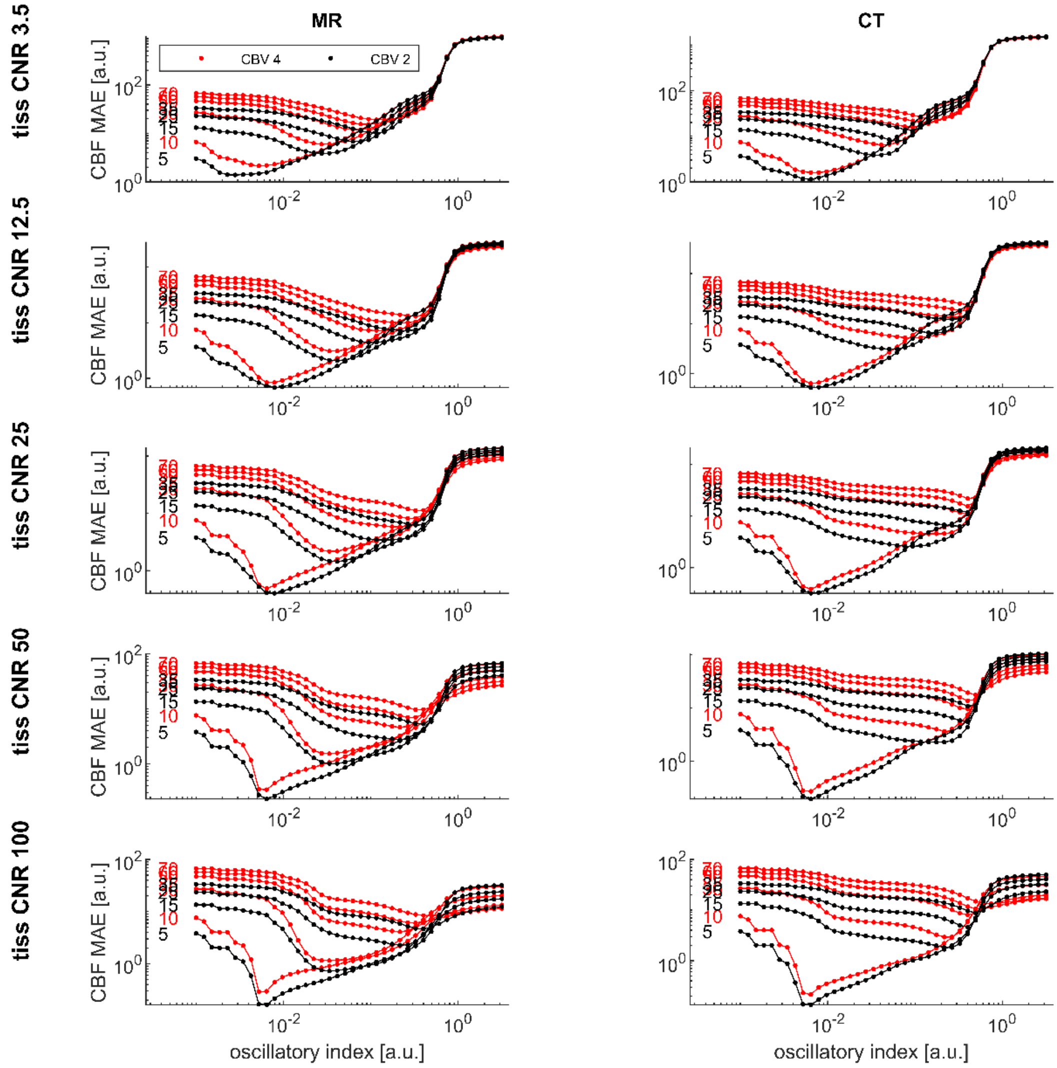

Sup. Fig. S1 MAE vs oscillation index (oSVD regularization) for tissue noise levels found in the in vivo data, CNR=3.5,12.5,25.50,100 and AIF CNR = 45, CBF levels [ml/100 ml/min] written out for identification purposes. Each datapoint represents 10 000 Monte Carlo noise iterations

Sup. Table S1. Optimal oSVD regularization for tissue DSC MRI with injection duration 3.2s and CNR=12.5, 50, 100, CTP with injection duration 12.5 s CNR=3.5, 25, 50 with AIF CNR = 45 for both modalities. Columns show the simulated CBF levels for two levels of CBV, corresponding to gray and white matter.

**Perfusion scenarios**				
CBV = 2 [ml/100ml]	5 [ml/100ml/min]	15 [ml/100ml/min]	25 [ml/100ml/min]	35 [ml/100ml/min]
DSC, CNR 12.5	0.0079	0.0412	0.0943	0.2649
DSC, CNR 50	0.0064	0.0335	0.2154	0.4005
DSC, CNR 100	0.0064	0.0335	0.2154	0.4005
CTP, CNR 3.5	0.0064	0.0335	0.0624	0.0943
CTP, CNR 25	0.0064	0.0767	0.3257	0.4005
CTP, CNR 50	0.0064	0.1425	0.4005	0.4005
CBV = 4 [ml/100ml]	10 [ml/100ml/min]	30 [ml/100ml/min]	50 [ml/100ml/min]	70 [ml/100ml/min]
DSC, CNR 12.5	0.0064	0.0412	0.1425	0.3257
DSC, CNR 50	0.0064	0.0335	0.2154	0.4005
DSC, CNR 100	0.0052	0.0335	0.2154	0.4005
CTP, CNR 3.5	0.0079	0.0412	0.0943	0.1425
CTP, CNR 25	0.0064	0.1425	0.4005	0.4005
CTP, CNR 50	0.0064	0.2649	0.4005	0.4924

Overlap and percentile statistics, supplements to Figures 5 and 6.

Sup. Table S2. Percentage of parameter estimates that fall below the ground truth value and overlap measures comparing relevant groups in Figure 5.

Simulation	Par	Simulated CBF level [ml/100ml/min]			
Figure 5		10	30	50	70
Percentage of parameter estimates below ground truth [%]					
CTP, CNR 25	CBF	57.5	79.8	82.7	95.5
	CBV	56.8	51.6	49.3	49.8
	MTT	48.7	22	29.1	7.7
DSC-MRI, CNR 25	CBF	51.3	73.7	71.6	95.8
	CBV	51.7	51.4	50.8	50.9
	MTT	53.9	30.2	32.2	6.1
CTP, CNR 50	CBF	41.7	87.1	77.6	97.3
	CBV	58.2	50.4	48.8	48.6
	MTT	64.2	25.7	29.3	6.7
DSC-MRI, CNR 50	CBF	37.2	70.2	73.7	97.1
	CBV	56	53.8	53.6	52.8
	MTT	68.8	35.4	30.4	3.4
Parameter estimate distribution overlap [%]					
CTP $T_{inj} = 12.5\ s$, CNR 25 vs. DSC-MRI $T_{inj} = 3.2\ s$, CNR 50	CBF	65.3	30.5	42	36.7
	CBV	65.4	65.4	64.7	65
	MTT	66.6	32	41.9	37.9
DSC-MRI $T_{inj} = 3.2\ s$, CNR 25 vs. DSC-MRI $T_{inj} = 3.2\ s$, CNR 50	CBF	64.5	70.3	75.6	79.7
	CBV	73.3	72.5	74.6	73.2
	MTT	63.2	68.7	74.9	79.5
CTP $T_{inj} = 12.5\ s$, CNR 25 vs. CTP $T_{inj} = 12.5\ s$, CNR 50	CBF	64.6	73.9	80.5	78.2
	CBV	66.7	68	68	66.9
	MTT	64.4	73.3	80.2	77.7

Sup. Table S3. Percentage of parameter estimates that fall below the ground truth value and overlap measures comparing relevant groups in Figure 6.

Simulation	Par	Simulated CBF level [ml/100ml/min]			
Figure 6		10	30	50	70
Percentage of parameter estimates below ground truth [%]					
CTP $T_{inj} = 12.5\ s$, CNR 3.5	CBF	58.1	79.1	78.3	76.2
	CBV	52.5	51.3	50.6	51.6
	MTT	46.7	27.5	26.8	27.4
DSC-MRI $T_{inj} = 3.2\ s$, CNR 3.5	CBF	65.8	74	68.9	86
	CBV	25.6	24.9	26.1	24.9
	MTT	18.4	16.1	21.7	9.6
CTP $T_{inj} = 12.5\ s$, CNR 12.5	CBF	65.3	68.5	89.7	96.7
	CBV	55.6	51.7	51	50
	MTT	41.3	35.2	24.9	5.7
DSC-MRI $T_{inj} = 3.2\ s$, CNR 12.5	CBF	48.2	74.4	73.1	93
	CBV	44.9	45.7	43.9	44.6
	MTT	50.4	27	28.8	8.8
Parameter estimate distribution overlap [%]					
CTP $T_{inj} = 12.5\ s$, CNR 3.5 vs. DSC-MRI $T_{inj} = 3.2\ s$, CNR 12.5	CBF	51.7	44.7	39.8	43.9
	CBV	58.5	60.2	59.2	60.7
	MTT	50.1	46.9	41.4	44.4
DSC-MRI $T_{inj} = 3.2\ s$, CNR 3.5 vs. DSC-MRI $T_{inj} = 3.2\ s$, CNR 12.5	CBF	38.3	49.4	57.8	60
	CBV	29.9	29	29.4	30.4
	MTT	27.3	36.2	45.1	46.1
CTP $T_{inj} = 12.5\ s$, CNR 3.5 vs. CTP $T_{inj} = 12.5\ s$, CNR 12.5	CBF	42.2	68.3	72.2	55.6
	CBV	33.8	33.1	33.9	34.5
	MTT	41.1	68	69.8	54.9

Illustrating differences between modality and CNR in simulations

Together with Figures 5 and 6, the Sup. Fig. S2-S5 illustrate the effect of the bolus injection duration for noise levels corresponding to small and large ROI as well as voxel-wise analysis, for both CBV levels 2 and 4 ml/100ml in CTP and DSC-MRI.

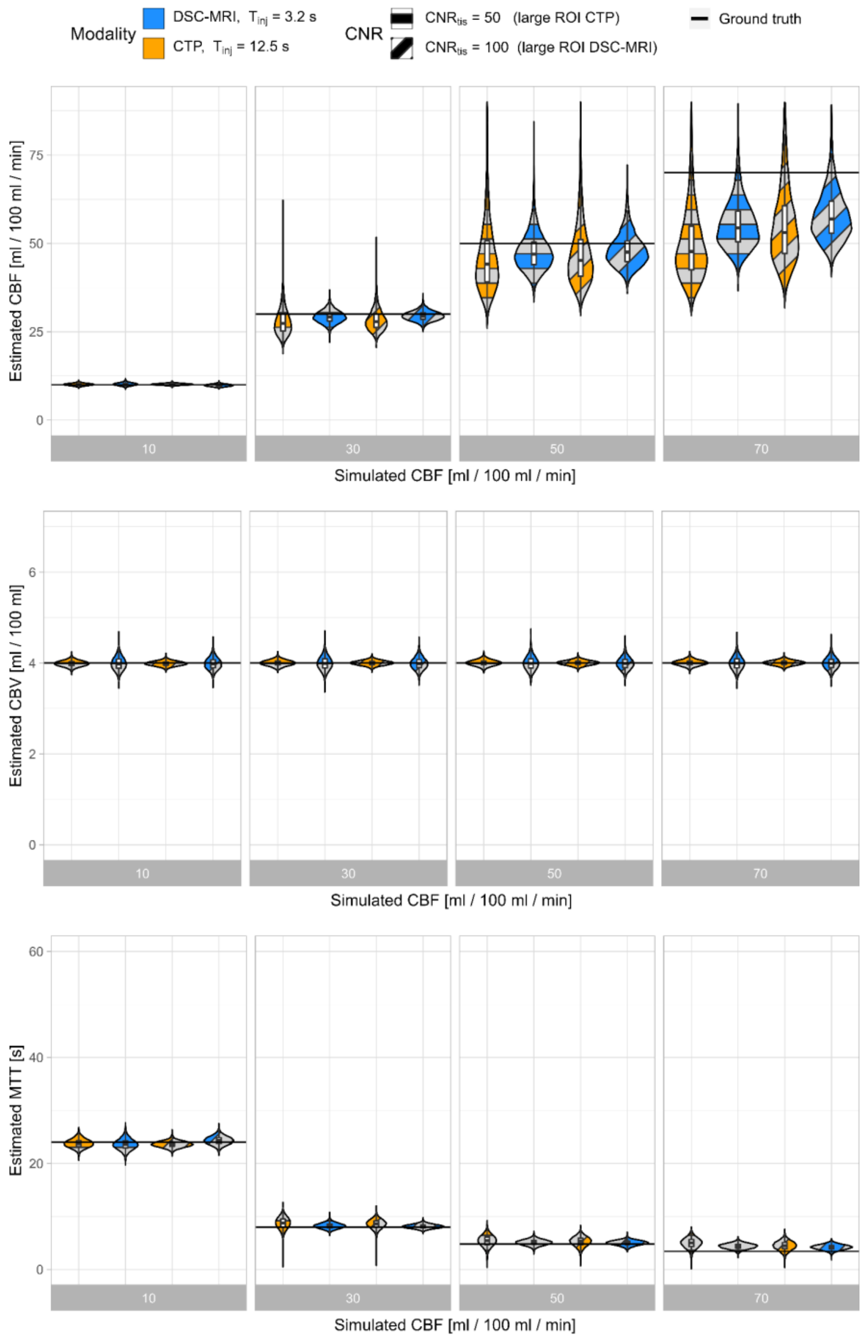

Sup. Fig. S2 Perfusion estimates from simulations with $\mathrm{CNR_{tiss}}$ = 50, 100 (orthogonal and diagonal pattern) and injection durations, $\mathrm{T_{inj}}$= 3.2, 12.5 s (orange, blue), typical for large ROI analysis in CTP and DSC-MRI, respectively. Combinations of the two levels of CNR and $\mathrm{T_{inj}}$ were evaluated yielding a total of four estimates per simulated perfusion level. The violin plots include the kernel density profile (violin sides) as well as a boxplot showing the median and inter quartile range. Estimates of CBV, CBF, and MTT estimated using oSVD with regularization selected individually based on the lowest resulting MAE for each simulated perfusion scenario, CBV 4 ml/100 ml and $\mathrm{CNR_{AIF}}$ = 45

Sup. Table S4. Percentage of parameter estimates that fall below the ground truth value and overlap measures comparing relevant groups in Sup. Fig. S2.

Simulation	Par	Simulated CBF level [ml/100ml/min]			
Sup. Fig. S2		10	30	50	70
Percentage of parameter estimates below ground truth [%]					
CTP $T_{inj} = 12.5\ s$, CNR 50	CBF	41.7	87.1	77.6	97.3
	CBV	58.2	50.4	48.8	48.6
	MTT	64.2	25.7	29.3	6.7
DSC-MRI $T_{inj} = 3.2\ s$, CNR 50	CBF	37.2	70.2	73.7	97.1
	CBV	56	53.8	53.6	52.8
	MTT	68.8	35.4	30.4	3.4
CTP $T_{inj} = 12.5\ s$, CNR 100	CBF	29.2	89.3	77.1	92.6
	CBV	60.2	49.5	47.5	47.5
	MTT	75.3	26	31	13.2
DSC-MRI $T_{inj} = 3.2\ s$, CNR 100	CBF	69.6	66.7	71.8	94.2
	CBV	56.3	55.1	55.1	54.4
	MTT	37.7	40	32.1	7
Parameter estimate distribution overlap [%]					
CTP $T_{inj} = 12.5\ s$, CNR 50 vs. DSC-MRI $T_{inj} = 3.2\ s$, CNR 100	CBF	56.1	33.2	44.3	36.7
	CBV	50.8	51.1	51.3	50.5
	MTT	58.5	33.3	42.9	36.5
DSC-MRI $T_{inj} = 3.2\ s$, CNR 50 vs. DSC-MRI $T_{inj} = 3.2\ s$, CNR 100	CBF	50.5	74.9	86.6	73.4
	CBV	87.3	87.6	87.4	87.6
	MTT	50.9	74.3	85.9	72.6
CTP $T_{inj} = 12.5\ s$, CNR 50 vs. CTP $T_{inj} = 12.5\ s$, CNR 100	CBF	69.6	74.5	84.2	63.6
	CBV	83.5	83.2	84.1	85
	MTT	70.8	74.9	84.4	63.6

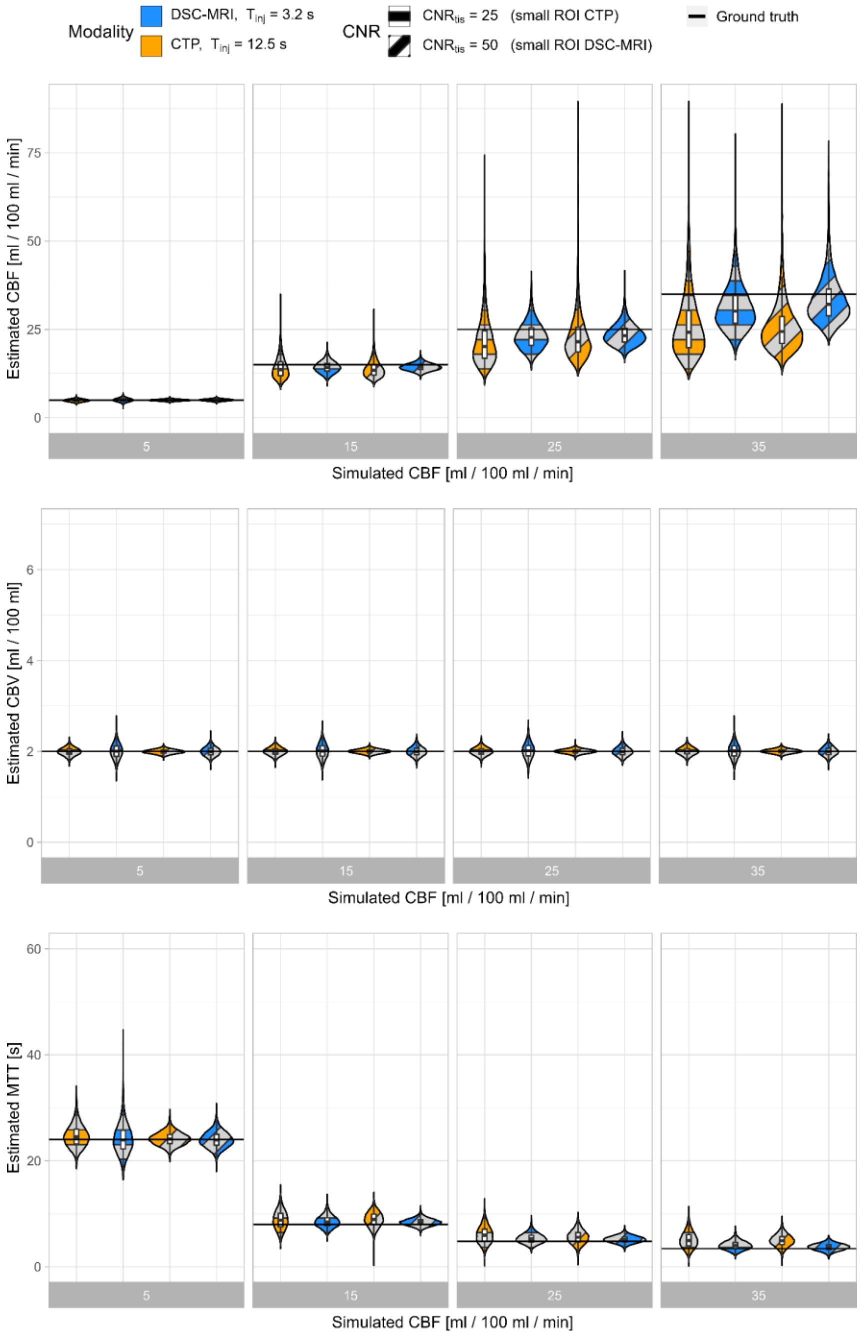

Sup. Fig. S3 Perfusion estimates from simulations with CNR_{tiss} = 25, 50 (orthogonal and diagonal pattern) and injection durations, T_{inj}= 3.2, 12.5 s (orange, blue), typical for small ROI analysis in CTP and DSC-MRI, respectively. Combinations of the two levels of CNR and T_{inj} were evaluated yielding a total of four estimates per simulated perfusion level. The violin plots include the kernel density profile (violin sides) as well as a boxplot showing the median and inter quartile range. Estimates of CBV, CBF, and MTT estimated using oSVD with regularization selected individually based on the lowest resulting MAE for each simulated perfusion scenario, CBV 2 ml/100 ml and CNR_{AIF} = 45

Sup. Table S5. Percentage of parameter estimates that fall below the ground truth value and overlap measures comparing relevant groups in Sup. Fig. S3.

Simulation	Par	Simulated CBF level [ml/100ml/min]			
Sup. Fig. S3		5	15	25	35
Percentage of parameter estimates below ground truth [%]					
CTP $T_{inj} = 12.5\ s$, CNR 25	CBF	64	68.1	90.2	92.2
	CBV	54.7	51.9	52.2	50.6
	MTT	41.7	34.3	25.3	15.6
DSC-MRI $T_{inj} = 3.2\ s$, CNR 25	CBF	50	68.3	72.2	78.7
	CBV	50.7	49	47.6	48.4
	MTT	53.8	35.9	31.6	27.5
CTP $T_{inj} = 12.5\ s$, CNR 50	CBF	57.4	83.6	76.7	92.2
	CBV	56.5	50.2	49.9	49.1
	MTT	48	27.2	29.1	10.3
DSC-MRI $T_{inj} = 3.2\ s$, CNR 50	CBF	49.8	75.5	72.7	70.7
	CBV	53.1	52.8	53.7	52.9
	MTT	53.3	30.8	30.2	33.3
Parameter estimate distribution overlap [%]					
CTP $T_{inj} = 12.5\ s$, CNR 25 vs. DSC-MRI $T_{inj} = 3.2\ s$, CNR 50	CBF	68.2	36.6	40.1	36.4
	CBV	85.8	85.8	86.3	84.3
	MTT	71.8	39	41	36.7
DSC-MRI $T_{inj} = 3.2\ s$, CNR 25 vs. DSC-MRI $T_{inj} = 3.2\ s$, CNR 50	CBF	58.8	64.2	76.9	74
	CBV	59.8	60.4	60.3	61.7
	MTT	58.2	62.5	73.2	72.3
CTP $T_{inj} = 12.5\ s$, CNR 25 vs. CTP $T_{inj} = 12.5\ s$, CNR 50	CBF	61.6	79.5	76.4	74.8
	CBV	58	58.1	55.6	57.2
	MTT	61.5	78.1	76.5	74.7

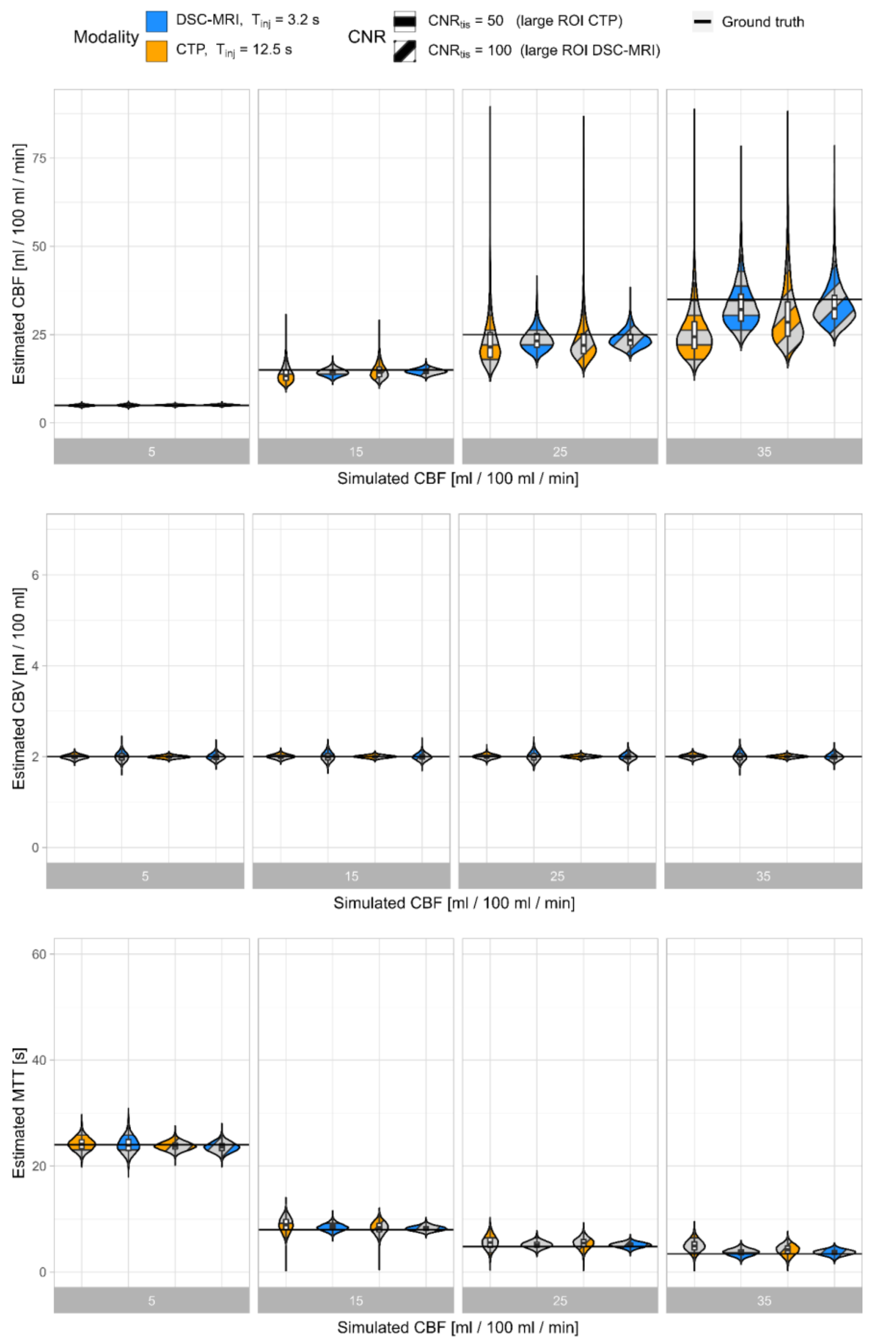

Sup. Fig. S4 Perfusion estimates from simulations with $CNR_{tiss} = 50, 100$ (orthogonal and diagonal pattern) and injection durations, T_{inj}= 3.2, 12.5 s (orange, blue), typical for large ROI analysis in CTP and DSC-MRI, respectively. Combinations of the two levels of CNR and T_{inj} were evaluated yielding a total of four estimates per simulated perfusion level. The violin plots include the kernel density profile (violin sides) as well as a boxplot showing the median and inter quartile range. Estimates of CBV, CBF, and MTT estimated using oSVD with regularization selected individually based on the lowest resulting MAE for each simulated perfusion scenario, CBV 2 ml/100 ml and $CNR_{AIF} = 45$

Sup. Table S6. Percentage of parameter estimates that fall below the ground truth value and overlap measures comparing relevant groups in Sup. Fig. S4.

Simulation	Par	Simulated CBF level [ml/100ml/min]			
Sup. Fig. S4		5	15	25	35
Percentage of parameter estimates below ground truth [%]					
CTP $T_{inj} = 12.5\ s$, CNR 50	CBF	57.4	83.6	76.7	92.2
	CBV	56.5	50.2	49.9	49.1
	MTT	48	27.2	29.1	10.3
DSC-MRI $T_{inj} = 3.2\ s$, CNR 50	CBF	49.8	75.5	72.7	70.7
	CBV	53.1	52.8	53.7	52.9
	MTT	53.3	30.8	30.2	33.3
CTP $T_{inj} = 12.5\ s$, CNR 100	CBF	43.8	77.4	78.6	79.3
	CBV	58.1	49.3	48.6	47.2
	MTT	63.7	39	28.2	25.2
DSC-MRI $T_{inj} = 3.2\ s$, CNR 100	CBF	36.5	71.3	75.1	71.9
	CBV	57.3	56.2	55.5	55.8
	MTT	69.5	35.7	28.6	32.6
Parameter estimate distribution overlap [%]					
CTP $T_{inj} = 12.5\ s$, CNR 50 vs. DSC-MRI $T_{inj} = 3.2\ s$, CNR 100	CBF	63.3	33.6	41.8	28.8
	CBV	67	66	64.3	65.3
	MTT	64.8	33.8	40.4	29.1
DSC-MRI $T_{inj} = 3.2\ s$, CNR 50 vs. DSC-MRI $T_{inj} = 3.2\ s$, CNR 100	CBF	64.3	70.2	78.8	85.8
	CBV	73.8	74	74.2	72.9
	MTT	62.9	69.1	77.3	84.8
CTP $T_{inj} = 12.5\ s$, CNR 50 vs. CTP $T_{inj} = 12.5\ s$, CNR 100	CBF	64.6	68.5	79.1	58.3
	CBV	67.7	67.8	68.1	68.7
	MTT	64.8	68.4	78.6	58.5

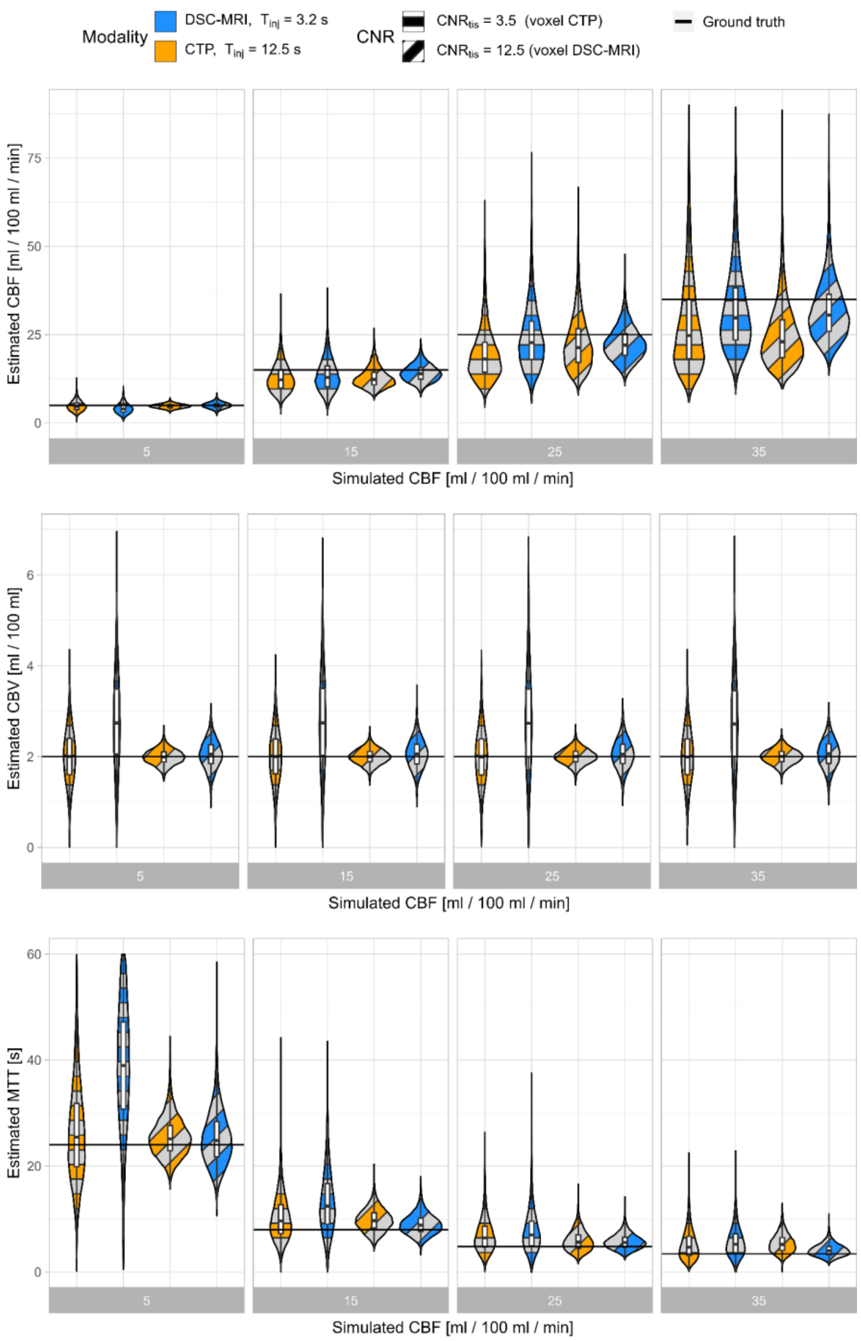

Sup. Fig. S5 Perfusion estimates from simulations with $CNR_{tiss} = 3.5, 12.5$ (orthogonal and diagonal pattern) and injection durations, T_{inj}= 3.2, 12.5 s (orange, blue), typical for voxel-wise analysis in CTP and DSC-MRI, respectively. Combinations of the two levels of CNR and T_{inj} were evaluated yielding a total of four estimates per simulated perfusion level. The violin plots include the kernel density profile (violin sides) as well as a boxplot showing the median and inter quartile range. Estimates of CBV, CBF, and MTT estimated using oSVD with regularization selected individually based on the lowest resulting MAE for each simulated perfusion scenario, CBV 2 ml/100 ml and $CNR_{AIF} = 45$

Sup. Table S7. Percentage of parameter estimates that fall below the ground truth value and overlap measures comparing relevant groups in Sup. Fig. S5.

Simulation	Par	Simulated CBF level [ml/100ml/min]			
Sup. Fig. S5		5	15	25	35
Percentage of parameter estimates below ground truth [%]					
CTP $T_{inj} = 12.5\ s$, CNR 3.5	CBF	60.5	75.7	84	76.6
	CBV	51.2	51	51.5	51.7
	MTT	45.4	34.9	27.4	31.4
DSC-MRI $T_{inj} = 3.2\ s$, CNR 3.5	CBF	77.5	68.8	63	68.1
	CBV	25.1	26.1	26.9	27
	MTT	20.3	21.8	26	23.2
CTP $T_{inj} = 12.5\ s$, CNR 12.5	CBF	66.5	81.1	70.5	99.6
	CBV	52.9	51.5	50.7	50.5
	MTT	39.1	22.3	34.4	13.3
DSC-MRI $T_{inj} = 3.2\ s$, CNR 12.5	CBF	53.6	67.5	74.7	71.5
	CBV	44.4	44.1	44.2	43.4
	MTT	45.2	34.4	28.6	32.4
Parameter estimate distribution overlap [%]					
CTP $T_{inj} = 12.5\ s$, CNR 3.5 vs. DSC-MRI $T_{inj} = 3.2\ s$, CNR 12.5	CBF	59.3	50.9	48.7	46.7
	CBV	55.1	56.8	55.1	54.9
	MTT	57	51.7	51.1	48
DSC-MRI $T_{inj} = 3.2\ s$, CNR 3.5 vs. DSC-MRI $T_{inj} = 3.2\ s$, CNR 12.5	CBF	43.8	52.7	60.3	68.8
	CBV	29.9	28.4	30.1	30.5
	MTT	21.3	34.6	43.8	44.5
CTP $T_{inj} = 12.5\ s$, CNR 3.5 vs. CTP $T_{inj} = 12.5\ s$, CNR 12.5	CBF	47.2	68.3	67.3	69.2
	CBV	33.8	34.2	33.1	33.8
	MTT	43.4	56.2	65.7	61.4

Separating the effects of signal model, CNR and injection duration in simulations

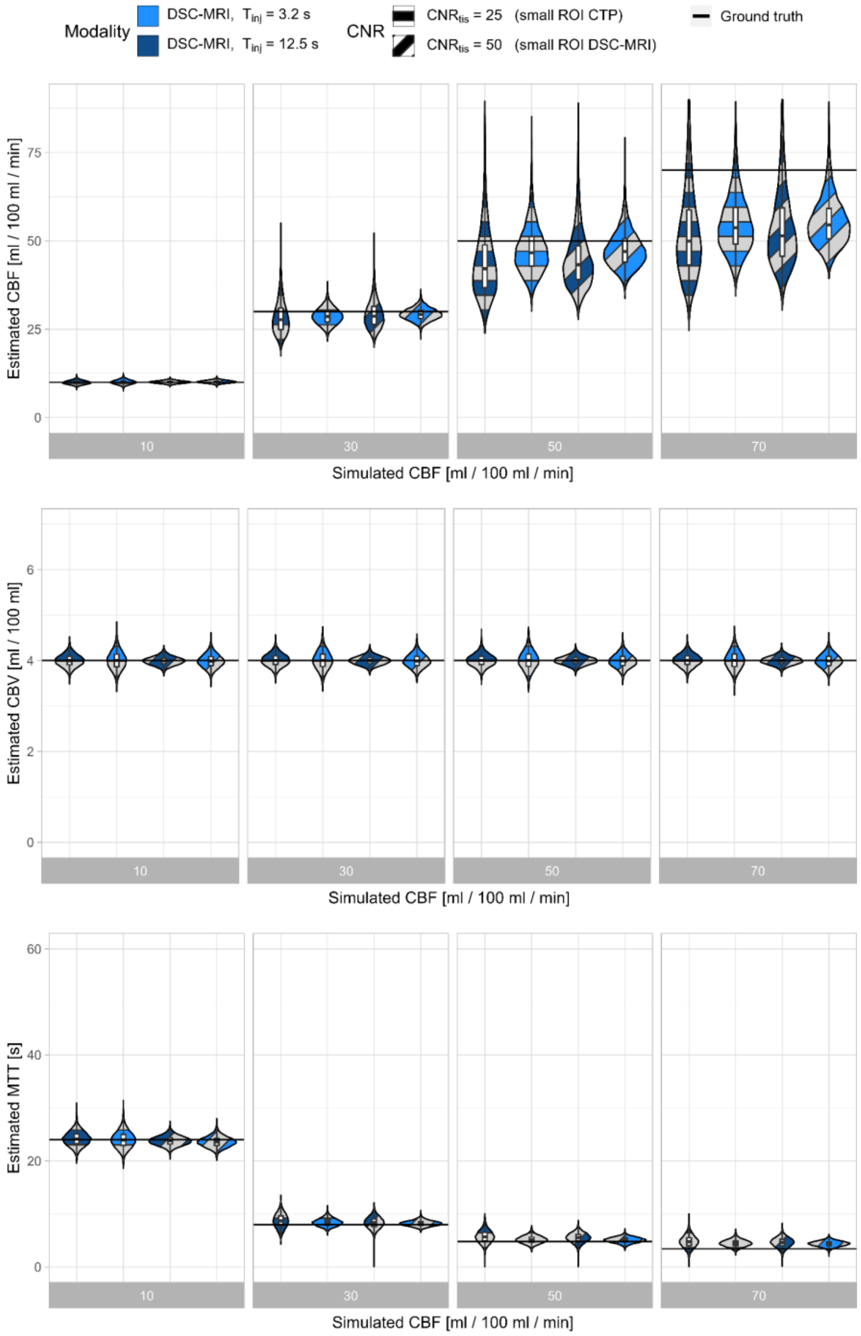

Sup. Fig. S6 Perfusion estimates from DSC-MRI simulations with T_{inj}= 3.2, 12.5 s (blue, dark blue), $CNR_{tiss} = 25, 50$ (orthogonal and diagonal pattern) and injection durations, typical for small ROI analysis in CTP and DSC-MRI, respectively. Combinations of the two levels of CNR and T_{inj} were evaluated yielding a total of four estimates per simulated perfusion level. The violin plots include the kernel density profile (violin sides) as well as a boxplot showing the median and inter quartile range. Estimates of CBV, CBF, and MTT estimated using oSVD with regularization selected individually based on the lowest resulting MAE for each simulated perfusion scenario, CBV 4 ml/100 ml and $CNR_{AIF} = 45$

Sup. Table S8. Percentage of parameter estimates that fall below the ground truth value and overlap measures comparing relevant groups in Sup. Fig. S6.

Simulation	Par	Simulated CBF level [ml/100ml/min]			
Sup. Fig. S6		10	30	50	70
Percentage of parameter estimates below ground truth [%]					
DSC-MRI $T_{inj} = 12.5\ s$, CNR 25	CBF	57	69.8	93.4	94.2
	CBV	51.9	49.8	50.1	49.5
	MTT	46.7	32.7	23.3	13
DSC-MRI $T_{inj} = 3.2\ s$, CNR 25	CBF	49.8	73.4	70.5	95.6
	CBV	51.7	51.4	51.4	50.9
	MTT	52.5	30.2	32.2	5.4
DSC-MRI $T_{inj} = 12.5\ s$, CNR 50	CBF	45.3	98.5	94.9	95.6
	CBV	56.2	53.2	52.4	52.8
	MTT	61.4	39	22.6	11.8
DSC-MRI $T_{inj} = 3.2\ s$, CNR 50	CBF	37.5	70.3	73	97
	CBV	55.8	54.8	53.8	53.8
	MTT	68.7	36.1	30.2	3.6
Parameter estimate distribution overlap [%]					
DSC-MRI $T_{inj} = 12.5\ s$, CNR 25 vs. DSC-MRI $T_{inj} = 3.2\ s$, CNR 50	CBF	61.9	35.6	43.7	46.9
	CBV	89.6	89.3	89.4	88.7
	MTT	62.7	37.4	43	46.5
DSC-MRI $T_{inj} = 3.2\ s$, CNR 25 vs. DSC-MRI $T_{inj} = 3.2\ s$, CNR 50	CBF	63.6	69.7	78.2	80.6
	CBV	73.6	73.1	73.3	73.9
	MTT	62.2	69.5	75.9	78.9
DSC-MRI $T_{inj} = 12.5\ s$, CNR 25 vs. DSC-MRI $T_{inj} = 12.5\ s$, CNR 50	CBF	65.6	55.7	74.9	79.9
	CBV	70.1	69.2	71.2	68.8
	MTT	63.6	73.2	76.3	80.1

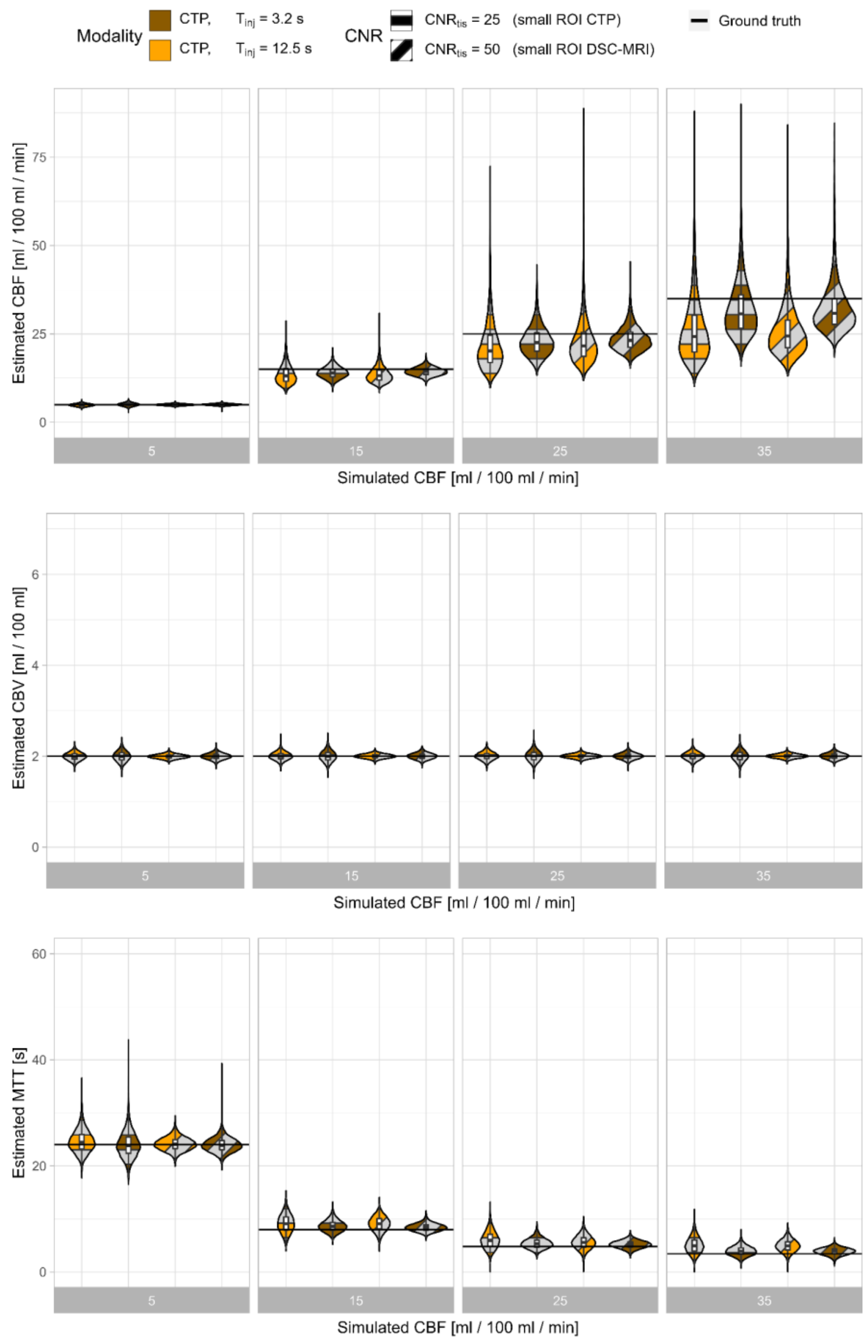

Sup. Fig. S7 Perfusion estimates from CTP simulations with T_{inj}= 3.2, 12.5 s (brown, orange), $CNR_{tiss} = 25, 50$ (orthogonal and diagonal pattern) and injection durations, typical for small ROI analysis in CTP and DSC-MRI, respectively. Combinations of the two levels of CNR and T_{inj} were evaluated yielding a total of four estimates per simulated perfusion level. The violin plots include the kernel density profile (violin sides) as well as a boxplot showing the median and inter quartile range. Estimates of CBV, CBF, and MTT estimated using oSVD with regularization selected individually based on the lowest resulting MAE for each simulated perfusion scenario, CBV 4 ml/100 ml and $CNR_{AIF} = 45$

Sup. Table S9. Percentage of parameter estimates that fall below the ground truth value and overlap measures comparing relevant groups in Sup. Fig. S7.

Simulation	Par	Simulated CBF level [ml/100ml/min]			
Sup. Fig. S7		5	15	25	35
Percentage of parameter estimates below ground truth [%]					
CTP $T_{inj} = 12.5\ s$, CNR 25	CBF	64.3	74.2	90	92
	CBV	54.8	52.1	51.3	50.8
	MTT	42.1	27.8	24.7	15.4
CTP $T_{inj} = 3.2\ s$, CNR 25	CBF	49.1	74.7	73.9	74.8
	CBV	52.7	52	50.6	50.2
	MTT	54.5	30.4	30.3	32.7
CTP $T_{inj} = 12.5\ s$, CNR 50	CBF	57.4	79.1	89.7	95.5
	CBV	56.3	51.6	50.6	49.7
	MTT	48.9	23.3	28.5	10.4
CTP $T_{inj} = 12.5\ s$, CNR 50	CBF	51.1	73.3	72.8	77.4
	CBV	55.2	50.5	52	49.2
	MTT	56.5	32	30.4	25.5
Parameter estimate distribution overlap [%]					
CTP $T_{inj} = 12.5\ s$, CNR 25 vs. CTP $T_{inj} = 3.2\ s$, CNR 50	CBF	60.6	38.1	45.2	38.6
	CBV	77.6	78.5	78.2	78.4
	MTT	62.1	39.8	44.6	38.5
CTP $T_{inj} = 3.2\ s$, CNR 25 vs. CTP $T_{inj} = 3.2\ s$, CNR 50	CBF	56.6	69.6	77.6	77.1
	CBV	58.8	58.4	57.5	58.8
	MTT	57.1	67.3	76.4	76
CTP $T_{inj} = 12.5\ s$, CNR 25 vs. CTP $T_{inj} = 12.5\ s$, CNR 50	CBF	62.1	78.5	77.9	75.5
	CBV	57.8	57.4	57.4	56.8
	MTT	60.8	78.8	76.8	75.4
